# Phytohormone metabolism in human cells: Cytokinins are taken up and interconverted in HeLa cell culture

**DOI:** 10.1096/fba.2018-00032

**Published:** 2019-03-12

**Authors:** Megan M. Aoki, Mark Seegobin, Anna Kisiala, Adam Noble, Craig Brunetti, R. J. Neil Emery

**Affiliations:** ^1^ Department of Biology Trent University Peterborough Ontario Canada; ^2^ Noblegen Peterborough Ontario Canada

**Keywords:** mammalian cells, mass spectrometry, N^6^‐benzyladenosine, N^6‐^isopentenyladenine

## Abstract

Cytokinins (CKs) encompass a group of phytohormones, known to orchestrate many critical processes in plant development. Excluding Archaea, CKs are pervasive among all kingdoms, but much less is reported about their metabolism beyond plants. Recent evidence from mammalian tissues indicates the presence of six additional CK forms beyond the previously identified, single mammalian CK, N^6^‐isopentenyladenosine (i^6^A). There is limited understanding of CK biosynthesis pathways in mammalian systems; therefore, human cervical cancer (HeLa) cells were used to further characterize CK processing by tracking the interconversion of CKs into their various structural derivatives in mammalian cells in a time‐course study. Through high‐performance liquid chromatography‐positive electrospray ionization‐tandem mass spectrometry (HPLC‐(+ESI)‐MS/MS), we document changes in the functional profiles of endogenous CKs in a human cell line following metabolism by HeLa cell cultures. The nucleotide CK fraction (iPRP) was found exclusively within the cell pellet (0.34 pmol/10^6^ cells), and the active free base (FB) form (iP) and riboside fraction (iPR) were found in greater abundance extracellularly (1.67 and 0.10 nmol/L respectively). For further confirmation, we demonstrate that HeLa cells metabolize an exogenously supplied CK, N^6^‐benzyladenosine (BAR). In the HeLa culture supernatant, a 12‐fold decrease in BAR concentration was observed within the first 24 hours of incubation accompanied by a fivefold increase in the FB form, N^6^‐benzyladenine (BA). These findings support the hypothesis that HeLa cells have the enzymatic pathways required for the metabolism of both endogenous and exogenous CKs.

**Abbreviations**
2MeSiP: 2‐methylthio‐N^6^‐isopentenyladenine
2MeSiPR: 2‐methylthio‐N^6^‐isopentenyladenosine
2MeSZ: 2‐methylthio‐zeatin
2MeSZR: 2‐methylthio‐zeatin riboside
BA: N^6^‐benzyladenine
BAR: N^6^‐benzyladenosine
BARP: N^6^‐benzyladenine‐9‐riboside‐5′ (either mono‐, di‐, or tri‐) phosphate
CK(s): cytokinin(s)
*c*Z: *cis*‐zeatin
*c*ZR: *cis*‐zeatin‐9‐riboside
*c*ZRP: *cis*‐zeatin‐9‐riboside‐5′ (either mono‐, di‐, or tri‐) phosphate
DMAPP: dimethylallyl pyrophosphate
DZ: dihydrozeatin
DZR: dihydrozeatin‐9‐ riboside
DZRP: dihydrozeatin‐9‐riboside‐5′ (either mono‐, di‐, or tri‐) phosphate
FB: free base
HeLa: human cervical cancer cell line
HPLC‐(+ESI)‐MS/MS: high‐performance liquid chromatography‐positive electrospray ionization tandem mass spectrometry
iP: N^6^‐isopentenyladenine
iPR (i^6^A in mammals): N^6^‐isopentenyladenosine
iPRP: N^6^‐isopentenyladenine‐9‐riboside‐5′ (either mono‐, di‐, or tri‐) phosphate
MEP: methylerythritol phosphate pathway
MRM: multiple reaction monitoring
MVA: mevalonate pathway
R: riboside
RP: nucleotide
SPE: solid phase extraction
*t*Z: *trans*‐zeatin
*t*ZR: *trans*‐zeatin‐9‐riboside
*t*ZRP: *trans*‐zeatin‐9‐riboside‐5′ (either mono‐, di‐, or tri‐) phosphate

## 1 INTRODUCTION

The cytokinins (CKs) encompass a group of phytohormones implicated in nearly all aspects of plant growth and development. CKs are thereby capable of whole‐plant organization where they have been documented in diverse roles such as cell division, flowering, organogenesis, and regulation of senescence, among many others.^1^, ^2^, ^3^ While CKs were once thought to be exclusive to plant taxa, they have now been discovered in all kingdoms of life—with the exception of Archaea.^4^

Structurally, CKs are adenine derivatives with an isoprenoid or aromatic side‐chain at the N^6^ terminus of the adenine. The N^6^‐side chain structure and configuration determine both the CK type and its activity. Of the naturally occurring CKs in plants, the isoprenoid‐type CKs, N^6^‐isopentenyladenine (iP), *trans*‐zeatin (*t*Z), *cis*‐zeatin (*c*Z), and dihydrozeatin (DZ), are the most common.^5^ Much less has been documented about aromatic‐type CKs, such as N^6^‐benzyladenine (BA).^6^, ^7^, ^8^, ^9^ The majority of naturally occurring CKs exist in distinctive structural derivatives or forms such as: free bases (FBs), ribosides (Rs), and nucleotides, or conjugates with glucose, xylose, or amino acid residues.^1^ In plants, the CK FBs are considered the most biologically active forms while glucose conjugates are considered to be either permanently inactive or reversible storage forms, depending on the glucose location.^7^, ^10^ These structural derivatives are central to the biosynthesis pathway and regulation of CK activity.

The commonly accepted CK biosynthesis pathway is based upon the model of isoprenoid biosynthesis in *Arabidopsis *summarized by Sakakibara,^5^ and later revised by Kamada‐Nobusada and Sakakibara.^11^ Within this model, two different pathways are involved in the synthesis of the four isoprenoid type CKs: the methylerythritol phosphate pathway (MEP; de novo pathway) and the mevalonate pathway (MVA; tRNA‐degradation pathway). iP, *t*Z, and DZ are predominately derived from the de novo biosynthesis, while *c*Z is believed to originate exclusively through the tRNA degradation.^12^ A group of enzymes, known as isopentenyltransferases (IPTs), initiate CK synthesis by N‐prenylation of the adenosine molecule at the N^6 ^terminus.^5^, ^13^, ^14^ In the MVA pathway, tRNA‐IPTs prenylate specific tRNA molecules at position A37; upon degradation, the tRNA‐derived CKs contribute to the pool of unbound CKs in the organism.^15^ When dimethylallyl pyrophosphate (DMAPP) acts as the prenyl donor, iP nucleotides (iPRPs; mono‐, di‐, and triphosphate) are the initial products.^5^, ^16^ iPRPs can be subsequently converted into various structural derivatives via the de novo or tRNA‐degradation pathway.

Sequence homology of the IPT genes (both adenylate and tRNA‐bound IPT) indicates that they are a family of highly conserved genes.^17^, ^18^ Adenylate‐IPT genes are accountable for the majority of endogenous CK within plants; however, tRNA‐IPTs also contribute to the pool of endogenous CKs, to a lesser degree when CKs bound to tRNA molecules are liberated, depending on the species.^15^, ^19^ tRNA‐IPT genes have been identified in a wide variety of organisms from single‐celled bacteria,^20^, ^21^ to complex, multicellular organisms, such as humans.^22^

Until recently, N^6^‐isopentenyladenosine (abbreviated i^6^A; synonymous to iPR in plants—the Rs fraction of the FB iP) was the only known mammalian CK—occurring either bound at position 37 to a subset of cytosolic and mitochondrial tRNAs^23^, ^24^, ^25^ or in its unbound form when it has degraded from tRNA.^26^, ^27^ In fact, tRNA degradation is thought to be the only source of CKs in mammalian systems.^28^ Bound to tRNA, i^6^A increases translation fidelity and efficiency.^25^ Currently, there are no other reported roles for this mammalian CK, in either its bound or free form.

Extensive profiling and mapping of CK biosynthesis pathways followed the discovery of natural CKs in plants 3; however, there has been no such focus on endogenous CKs in any mammalian system. CKs have been gaining much attention in mammalian studies, mostly for their potential therapeutic roles as anticancer agents when applied exogenously to in vitro cell cultures.^29^, ^30^ Much emphasis has been placed on documenting novel CKs that exert cytotoxicity on a wide range of mammalian cancer cell lines.^30^, ^31^ Through these studies, it is evident that the structural derivatives of the CK are essential to their specific anticancer activity—with the Rs fractions exerting the most cytotoxic effects. While insight into the roles of CKs in mammalian cells is gained through these studies, much remains to be understood about the in vivo metabolism and function of endogenous mammalian CKs.

Recently, Seegobin et  al^32^ unequivocally detected the presence of seven CK forms in a wide selection of canine tissues: iPR, iPRP (mono‐, di‐, and triphosphate), *cis*‐zeatin‐9‐riboside (*c*ZR), *cis*‐zeatin‐9‐riboside‐5′ (either mono‐, di‐, or tri‐) phosphate (*c*ZRP), 2‐methylthio‐N^6^‐isopentenyladenine (2MeSiP), 2‐methylthio‐N^6^‐isopentenyladenosine (2MeSiPR), and 2‐methylthio‐zeatin riboside (2MeSZR) by mass spectrometry. This study thereby provided evidence of at least six new CK forms present in mammalian systems, in addition to the single, previously reported form—iPR. However, it is unclear the extent to which mammalian systems have the enzymatic pathways responsible for the interconversion of CKs to and from various structural derivatives that are either endogenously synthesized or exogenously received through interaction with bacteria, fungi, or from other sources.

To determine whether mammalian cells have the capacity to interconvert both endogenous and exogenous CKs, human cervical cancer (HeLa) cells were employed as a model system. This time‐course study included monitoring various endogenous and exogenous CK derivatives to identify whether HeLa cultures possess the cellular capacity to modify CKs in a similar manner as previously described for plants and microorganisms. Through high‐performance liquid chromatography‐electrospray ionization‐tandem mass spectrometry (HPLC‐(ESI+)‐MS/MS), we demonstrate the ability of HeLa cells to convert CKs into their various structural derivatives. This work documents the metabolism of endogenous iP‐type CKs and the capacity for metabolizing an exogenously supplied (non‐endogenously occurring) aromatic CK, N^6^‐benzyladenosine (BAR).

## 2 MATERIALS & METHODS

### 2.1 Cell line, chemicals, and reagents

Henrietta Lack human cervical cancer cells were obtained from the American Type Culture Collection (ATCC; Manassas, VA). Dulbecco's modified eagle's medium (DMEM) and other necessary supplements were obtained from Hyclone (Ottawa, ON, Canada). The cells were cultured in DMEM and supplemented with 10% fetal bovine serum, 2 mmol/L L‐glutamine, 100 µg/mL penicillin, and 100 µg/mL streptomycin. BAR (OlChemim Ltd., Olomouc, Czech Republic) was dissolved in a minimal volume of 1 mol/L NaOH and diluted with methanol (CH_3_OH) to prepare a stock solution for cell culture treatments.

### 2.2 Cell culture conditions and sampling for CK analysis

The HeLa cells were cultured in an incubator under standard conditions (37°C, 5% CO_2_). HeLa cells were seeded at a cell density of 2.5 × 10^6^ in T175 flasks with 50 mL of medium at time zero. A medium blank was used as a control and a total of three replicates were used for each treatment. The flasks were subsequently incubated for 72 hours. About 5 mL of supernatant or control medium was collected using sterile procedures from each flask for hormone extraction at the 24‐, 48‐, and 72‐hour time‐points. For each of the supernatant samples collected, the collection tubes were centrifuged (5 minutes at 4696 xg) and the supernatant was carefully transferred to a new sterile 15 mL tube to avoid contamination from potential living or dead cell debris. Additionally, at the 72‐hour time‐point, the cells were pelleted following trypsinization (5 minutes at 4696 xg), washed twice with phosphate‐buffered saline (PBS), and re‐pelleted. An aliquot was collected for counting with a hemocytometer prior to re‐pelleting. The fresh weight for each cell pellet sample was obtained and recorded prior to cell counting by suctioning off all remaining liquid following centrifugation and weighing the cell pellet mass in pre‐weighed 1.5 mL centrifuge tubes on an analytical scale (0.1 g readability; Sartorius Practum 224‐1S). All the collected supernatant and pellet samples were immediately placed at −20°C and stored until CK metabolites were extracted for mass spectrometry analysis.

For the BAR‐treatment study, HeLa cells were seeded at a density of 2.5 × 10^6^ cells per T175 flask filled with 60 mL of DMEM medium. Medium treated with 10^−6 ^mol/L BAR is referred to as the treated medium blank and served as a no cells control. Three biological replicates were used for each treatment. At 6, 12, 24, 48, and 72 hours following treatment, 5 mL of culture supernatant or blank medium was collected from each flask for CK analysis, following the same procedure as described above and stored immediately at −20°C prior to hormone extraction. At the 72‐hour time point, the cells were pelleted following trypsinization and counted using a hemocytomer. Prior to cell counting with a hemocytometer, the cells were stained with a 0.25% trypan blue solution (Invitrogen, Carlsbad, CA) to assess the quantity of live and dead cells per to total number of cells counted.

### 2.3 Cytokinin extraction and purification

A modified protocol of hormone extraction and purification utilizing conditions established to minimize enzymatic activities causing CK nucleotide degradation and CK isomerization as described by Quesnelle and Emery^33^ and Farrow and Emery^34^ was used for processing medium blanks, supernatant, and pellet samples from both experiments. One milliliter of extraction buffer Bieleski #2 (CH_3_OH:H_2_0:HCO_2_H [15:4:1, v/v/v]) was added to each liquid sample. The samples were consecutively spiked with 10 ng of each of the deuterated internal standard CKs (OlChemim Ltd.; Table 1) and were allowed to passively extract overnight at −20°C. The following day, an additional 1 mL of the extraction buffer was added to the samples, and the combined supernatants were evaporated in a speed vacuum concentrator (Savant SPD111V, UVS400; Thermo Fisher Scientific, Waltham, MA) at ambient temperature. The cell pellet samples followed the same protocol but were homogenized in a ball mill (RetschMM300, Haan, Germany; 25 Hz, 3 minutes) at 4°C with zirconium oxide grinding beads (Comeau Technique Ltd., Vaudreuil‐Dorion, QC, Canada) after the addition of 1 mL of extraction buffer and internal CK standards.

**Table 1:** Endogenous and ^2^H‐labelled cytokinins (CK) scanned for by liquid chromatography‐positive electrospray ionization‐tandem mass spectrometry (HPLC‐(ESI+)‐MS/MS) in HeLa cell pellets and supernatants. Labelled internal standards (OlChemim Ltd.; Olomouc, Czech Republic) were used to identify and quantify CKs

Endogenous CK Fractions	^2^H‐labelled internal standard
Nucleotides (RP)	
*trans*‐zeatin riboside‐5′‐monophosphate (*t*ZRP)	^2^H_6_[9RMP]DZR
*cis*‐zeatin riboside‐5′‐monophosphate (*c*ZRP)	
Dihydrozeatin riboside‐5′‐monophosphate (DZRP)	
*N* ^6^‐benzyladenosine‐5′monophosphate (BARP)	
*N* ^6^‐isopentyladenosine‐5′monophosphate (iPRP)	^2^H_6_[9RMP]iP
Ribosides (R)	
*trans*‐zeatin riboside (*t*ZR)	^2^H_5_[9R]Z
*cis*‐zeatin riboside (*c*ZR)	
Dihydrozeatin riboside (DZR)	^2^H_3_[9R]DZ
*N* ^6^‐isopentyladenosine (iPR)	^2^H_6_[9R]iP
*N* ^6^‐benzyladenosine (BAR)	^2^H_7_[9R]BA
Free bases (FB)	
*trans*‐zeatin (*t*Z)	^2^H_3_DZ
*cis*‐zeatin (*c*Z)	
Dihydrozeatin (DZ)	
*N* ^6^‐isopentyladenine (iP)	^2^H_6_iP
*N* ^6^‐benzyladenine (BA)	^2^H_7_BA
*ortho*‐topolin (*o*T)	^15^N_4_ *o*T
Kinetin (KIN)	^15^N_4_K
Glucosides (GLUC)	
*trans*‐Zeatin‐O‐glucoside (*t*ZOG)	^2^H_5_ *t*ZOG
*cis*‐Zeatin‐O‐glucoside (*c*ZOG)	
Dihydrozeatin‐O‐glucoside (DZOG)	^2^H_7_DZOG
*trans*‐Zeatin‐O‐glucoside riboside (*t*ZOGR)	^2^H_5_ *t*ZROG
*cis*‐Zeatin‐O‐glucoside riboside (*c*ZOGR)	
Dihydrozeatin‐O‐glucoside riboside (DZOGR)	^2^H_7_DZROG
*trans*‐Zeatin‐7‐glucoside (*t*Z7G)	^2^H_5_ *t*Z7G
*trans*‐Zeatin‐9‐glucoside (*t*Z9G)	^2^H_5_ *t*Z9G
*cis*‐Zeatin‐9‐glucoside (*c*Z9G)	
Dihydrozeatin‐9‐glucoside (DZ9G)	^2^H_3_DZ9G
Methylthiols (2MeS)	
2‐Methylthio‐*trans*‐zeatin (2MeSZ)	^2^H_5_MeSZ
2‐Methylthio‐*trans*‐zeatin riboside (2MeSZR)	^2^H_5_MeSZR
2‐Methylthio‐*N* ^6^‐isopentenyladenine (2MeSiP)	^2^H_6_MeSiP
2‐Methylthio‐*N* ^6^‐isopentenyladenosine (2MeSiPR)	^2^H_6_MeSiPR

Extraction residues from both the supernatant and the pellet samples were reconstituted in 1 mL of 1 mol/L formic acid (HCO_2_H, pH 1.4) to ensure complete protonation of all CKs. Each extract was subjected to solid phase extraction (SPE) on a mixed mode, reversed‐phase, cation‐exchange cartridge (MCX 6cc; Canadian Life Sciences, Peterborough, ON, Canada). Cartridges were activated with 5 mL of CH_3_OH and equilibrated to initial conditions with 5 mL of 1 mol/L HCO_2_H. Each sample was loaded and allowed to pass through the column by gravity and the column was then washed with 5 mL of 1 mol/L HCO_2_H, followed by 5 mL of CH_3_OH. The nucleotide fraction was eluted first using 5 mL of 0.35 mol/L NH_4_OH to be processed separately from the other CK fractions, which are retained on the column owing to their cationic properties.^35^ FBs, Rs, methylthiols, and glucosides were sequentially eluted using 5 mL of 0.35 mol/L NH_4_OH in 60% CH_3_OH. Both eluted fractions were evaporated in a speed vacuum concentrator at ambient temperature and stored at −20°C.

Since CK nucleotides cannot be directly analyzed by our LC‐MS/MS method, the separately eluted anionic nucleotide samples were dephosphorylated using three units of alkaline phosphatase (alkaline phosphatase calf intestine; New England BioLabs, Whitby, ON, Canada) in 1mL  of 0.1M ethanolamine‐HCL (pH 10.4) for 12 hours at 37°C.^36^ The resulting Rs were evaporated in a speed vacuum concentrator at ambient temperature. This detection method of the nucleotide fraction reflects the pooled contribution of mono‐, di‐, and triphosphates where the isopentenyl (iPRP) or hydroxylated moiety (*c*ZRP, *t*ZRP, and dihydrozeatin‐9‐riboside‐5′ (either mono‐, di‐, or tri‐) phosphate) can be transferred to AMP, ADP, or ATP. Samples were reconstituted in 1.5 mL Milli‐Q H_2_O for further purification on a reversed‐phase C_18_ column (C_18_ 6cc; Canadian Life Sciences). Columns were activated using 3 mL of CH_3_OH and equilibrated with 6 mL of Milli‐Q H_2_O. Samples were loaded onto the C_18 _cartridge and were allowed to pass through the column by gravity. The residue was washed with 3 mL of Milli‐Q H_2_O and the Rs were eluted using 1.25 mL of 80% CH_3_OH:H_2_O (80:20 v/v). All sample eluents were evaporated and stored at −20°C until further processing.

Prior to HPLC‐(ESI+)‐MS/MS analysis, the extracted CK fractions were reconstituted in 1.5 mL of initial mobile phase conditions (95:5 H_2_O:C_2_H_3_OH [acetonitrile] with 0.08% CH_3_CO_2_H [acetic acid]). Samples were transferred to glass autosampler vials and stored at −20°C until analysis.

### 2.4 Cytokinin quantification

Human cervical cancer cell pellet, supernatant, and medium blank samples were analyzed by high‐performance liquid chromatography‐positive electrospray ionization‐tandem mass spectrometry (HPLC‐(ESI+)‐MS/MS; Shimadzu LC‐10ADvp HPLC (Columbia, MD, USA) coupled with a QTrap 5500 mass spectrometer (Sciex Applied Biosystem, Concord, ON, Canada) with a turbo V‐spray ionization source. HPLC‐(ESI+)‐MS/MS methods and multiple reaction monitoring (MRM) channels, specific for each CK, were carried out as described by Farrow and Emery.^34^ A 20 µL sample aliquot was injected on a reversed‐phase C_18_ column (Kinetex 2.6u C_18_ 100 A, 2.1 × 50mm ; Phenomenex, Torrance, CA), and CKs were eluted with an increasing gradient of solvent B (0.08% CH_3_CO_2_H in C_2_H_3_N) mixed with solvent A (0.08% CH_3_CO_2_H in H_2_O), at a flow rate of 0.4 mL/min. The HPLC method for CK separation involved a multistep gradient as follows: starting conditions were 5% solution B, which increased linearly to 10% over a 2‐minute window; over the following 6.5 minutes, solution B increased to 95% and was held constant for 1.5 minutes before returning back to starting conditions for 5 minutes of column re‐equilibration.

### 2.5 Statistical analysis

The mass spectrometry data were processed using Analyst v. 1.6.2 software (AB Sciex, Concord, ON, Canada). Quantification was achieved through isotope dilution analysis based on direct comparison of the analyte peak area to that of the recovered ^2^H‐labeled internal standards (Table 1).^34^ The analysis of *cis *isomers of the zeatin‐type CKs was evaluated relative to the recovery of labeled *t*Z‐type CKs and the retention time of endogenous *c*Z‐type CKs. Furthermore, the reported levels of 2MeSZ and 2MeSZR reflect a pool of *cis*‐ and *trans*‐methylthiolated (2MeS) zeatin. Concentrations of CK types in the supernatant samples are expressed as nmol/L, whereas pellet concentrations are expressed as pmol/10^6^ cells. Statistical analysis of phytohormone data was conducted using an ANOVA with a Tukey's post hoc test. In the statistical analyses, all significant differences refer to a *P*‐value of <0.05 (*n* = 3). All statistical analyses were carried out using PRISM v.6.0 (GraphPad Software, Inc, San Diego, CA).

## 3 RESULTS

### 3.1 Endogenous CK profiling in HeLa cells

Human cervical cancer cells were cultured over a 72‐hour period during which samples were collected for CK analysis every 24 hours. Medium blank, supernatant, and cell pellet samples were analyzed to determine the presence of any endogenous CK production in the pellet samples and/or CK secretion into the culture medium. Over 30 different CKs (known to naturally occur in plant systems) were scanned for by HPLC‐(ESI+)‐MS/MS (Table 1). In the DMEM medium blank, iPR, 2MeSZ, and 2MeSiP were detected with average concentrations of 0.38, 0.32, and 0.22 nmol/L respectively. The levels of CKs detected in the medium blank samples were stable throughout the incubation period; therefore, any changes observed upon addition of HeLa cells were considered of biological origin. Data on other common mammalian cell culture medium types (MEM and RPMI) share similar background CK analytes (Aoki and Emery, unpublished).

In the HeLa supernatant samples, three CK types in addition to the CKs present in the medium blank were measured totalling six CK types detected in the HeLa cell culture supernatant samples: iP, iPR, 2MeSZ, 2MeSiP, 2MeSZR, and 2MeSiPR (Table 2). We did not observe a proportional doubling in CK concentration in the supernatant samples throughout each 24‐hour sampling interval, corresponding to the approximate 24‐hour doubling time of HeLa cells; however, all CK forms did increase and the highest concentrations were consistently detected at the 72‐hour time point for each CK form (fold changes from 24 to 72 hours were as follows: iP, 3.48; iPR, 3.33; 2MeSZ, 7.48; 2MeSiP, 3.27; and 2MeSZR, 31.67). This concurs with the idea of CK secretion by HeLa cells and accumulation in the supernatants throughout the incubation period. Interestingly, iP, 2MeSiPR, and 2MeSZR were only found in the medium upon culturing with HeLa cells. Furthermore, the CK FB analyte, iP, significantly increased in concentration throughout the incubation period from 0.48 nmol/L at the 24‐hour time point to 1.67 nmol/L at the 72‐hour time point.

**Table 2:** Concentrations of cytokinin (CK) forms detected extracellularly in the untreated HeLa supernatants (nmol/L) and intracellularly within the corresponding cell pellets (pmol/10^6^ cells). Presented values are means ± SE (n = 3). A separate ANOVA was performed for each CK form for the supernatant samples with time as the dependent variable followed by Tukey's post hoc analysis

Sampling time (h)	CK concentration (nmol/L, supernatant; pmol/10^6^ cells, pellet)						
	iP	iPR	2MeSZ	2MeSiP	2MeSZR	2MeSiPR	iPRP
24	0.48 ± 0.02^b^	0.03 ± 0.01^NS^	0.29 ± 0.03^b^	0.41 ± 0.30^NS^	0.03 ± 0.02^NS^	n.d.	n.d.
48	0.61 ± 0.04^b^	0.05 ± 0.01	0.34 ± 0.10^b^	0.56 ± 0.39	0.12 ± 0.12	0.10 ± 0.02^NS^	n.d.
72	1.67 ± 0.05^a^	0.10 ± 0.05	2.17 ± 1.86^a^	1.34 ± 0.64	0.95 ± 0.89	0.20 ± 0.08	n.d.
Pellet (72)	0.02 ± 0.003	0.05 ± 0.02	0.12 ± 0.04	0.08 ± 0.02	0.02 ± 0.02	0.13 ± 0.04	0.34 ± 0.14

Values with the same letters are not significantly different (*P* < 0.05).

NS indicates lack of significant difference in CK concentrations between all time points.

The six CK forms found in supernatant samples were also detected in the cell pellet, with the addition of iPRP (Table 2). Interestingly, among CKs detected in the pellet, iPRP was present in the highest concentration (0.34 pmol/10^6^ cells; Table 2). iP‐type CKs (FB, R, and RP fractions) were the most highly represented (55%) in the cell pellets. Notably, an inverse relationship was observed between the concentration of iP and iPR in the pellet and the supernatant samples. iPR was 2.5‐fold greater in concentration than iP intracellularly, whereas in the supernatant samples, iP was 16‐fold greater in concentration versus iPR (Table 2).

### 3.2 HeLa cells metabolize an exogenously supplied CK

To test if mammalian cells can metabolize an exogenously supplied CK into its various structural derivatives, HeLa cells were cultured in medium supplemented with three different concentrations of BAR (10^−12^, 10^−9^, and 10^−6^ mol/L) over a 72‐hour incubation period. BA structural derivatives (FB, R, and RP) have not been documented in mammalian systems, nor have we detected their presence in untreated cultures; therefore, this aromatic CK is a suitable candidate for the metabolism analysis. Five sampling points were used to analyze changes in CK concentration throughout the 72‐hour incubation period: 6‐, 12‐, 24‐, 48‐, and 72 hours. The HeLa cells were pelleted only at the 72‐hour time point, once the flasks were confluent and had enough cell mass for homone extraction and analysis via mass spectrometry.

In the treated medium blank, six CK types were detected upon the addition of 10^‐6^ M BAR solution: BA, *c*Z, BAR, iPR, BARP, and *t*ZRP (Table 3). Direct injection of the BAR stock solution into the mass spectrometer revealed that these additional CKs (*c*Z, BA, iPR, BARP, and *t*ZRP) were from the BAR source and were therefore not above background levels in the samples. Moreover, no significant differences were observed in the concentrations of the analytes detected in the BAR‐treated, no cell control samples throughout the incubation period, indicating there was no degradation of the compound or interaction with the medium components throughout the time‐course study (Table 3).

**Table 3:** Cytokinin (CK) forms and concentrations detected in the 10^−6^ mol/L BAPR treated medium blank, the 10^−9^ and 10^−6^ mol/L BAPR treated HeLa supernatants (nmol/L), and intracellularly in the corresponding treated cell pellets (pmol g/FW). Presented values are means ± SE (n = 3). A separate ANOVA was performed for each CK form for the supernatant samples with time as the dependent variable followed by Tukey's post hoc analysis

Treatment	Sampling time (h)	CK concentration (nmol/L, supernatant; pmol/10^6^ cells, pellet)							*c*ZRP	*t*ZRP
		BA	iP	*c*Z	BAR	iPR	BARP	iPRP		
Treated medium (10^−6^ mol/L BAPR, no cell) control	6	1.72 ± 0.39^NS^	n.d.	0.39 ± 0.01^NS^	518.16 ± 24.77^NS^	0.27 ± 0.03^NS^	0.12 ± 0.03^NS^	n.d.	n.d.	0.10 ± 0.03^NS^
	12	1.51 ± 0.25	n.d.	0.39 ± 0.04	474.45 ± 9.22	0.26 ± 0.01	0.14 ± 0.02	n.d.	n.d.	0.10 ± 0.06
	24	1.77 ± 0.24	n.d.	0.41 ± 0.06	479.84 ± 31.84	0.27 ± 0.01	0.14 ± 0.03	n.d.	n.d.	0.21 ± 0.09
	48	1.49 ± 0.20	n.d.	0.43 ± 0.04	449.26 ± 9.72	0.28 ± 0.04	0.16 ± 0.01	n.d.	n.d.	0.12 ± 0.06
	72	2.41 ± 0.19	n.d.	0.33 ± 0.02	496.21 ± 1.06	0.26 ± 0.002	0.11 ± 0.03	n.d.	n.d.	0.09 ± 0.07
10^−9 ^mol/L BAR	6	1.47 ± 0.19^b^	0.14 ± 0.02^b^	0.58 ± 0.13^NS^	0.52 ± 0.09^NS^	0.13 ± 0.03^NS^	0.92 ± 0.82^NS^	n.d.	n.d.	1.09 ± 0.73^ab^
	12	1.57 ± 0.14^ab^	0.24 ± 0.05^b^	0.61 ± 0.22	0.25 ± 0.03	0.13 ± 0.03	0.69 ± 0.45	n.d.	n.d.	1.92 ± 1.52^a^
	24	1.71 ± 0.10^ab^	0.22 ± 0.01^b^	0.59 ± 0.12	0.34 ± 0.04	0.15 ± 0.02	0.30 ± 0.06	n.d.	n.d.	0.53 ± 0.15^b^
	48	1.64 ± 0.02^ab^	0.38 ± 0.004^b^	0.72 ± 0.18	0.25 ± 0.02	0.22 ± 0.03	0.24 ± 0.11	n.d.	n.d.	0.72 ± 0.21^ab^
	72	1.99 ± 0.25^a^	1.27 ± 0.03^a^	0.48 ± 0.04	0.18 ± 0.04	0.29 ± 0.01	0.08 ± 0.04	n.d.	n.d.	0.31 ± 0.16^b^
	Pellet (72)	0.006 ± 0.006	n.d.	n.d.	0.003 ± 0.0006	0.006 ± 0.0004	n.d.	0.20 ± 0.03	0.004 ± 0.0008	0.001 ± 0.0002
10^−6 ^mol/L BAR	6	121.97 ± 10.38^c^	0.03 ± 0.02^c^	0.46 ± 0.08^NS^	527.61 ± 27.56^a^	0.26 ± 0.08^NS^	0.98 ± 0.82^NS^	n.d.	n.d.	0.45 ± 0.27^NS^
	12	270.93 ± 7.06^b^	0.11 ± 0.01^bc^	0.27 ± 0.14	406.27 ± 63.60^b^	0.15 ± 0.01	0.26 ± 0.15	n.d.	n.d.	0.37 ± 0.17
	24	577.00 ± 23.85^a^	0.20 ± 0.02^bc^	0.28 ± 0.16	43.57 ± 8.30^c^	0.04 ± 0.02	0.18 ± 0.11	n.d.	n.d.	0.55 ± 0.26
	48	520.29 ± 27.00^a^	0.31 ± 0.06^b^	0.13 ± 0.07	33.72 ± 6.90^c^	0.02 ± 0.02	0.22 ± 0.04	n.d.	n.d.	0.45 ± 0.07
	72	514.50 ± 44.18^a^	1.29 ± 0.24^a^	0.34 ± 0.10	23.65 ± 3.31^c^	0.06 ± 0.03	0.69 ± 0.25	n.d.	n.d.	0.89 ± 0.09
	Pellet (72)	0.03 ± 0.005	n.d.	n.d.	0.04 ± 0.005	0.005 ± 0.0004	n.d.	1.09 ± 0.77	0.004 ± 0.00003	0.00098 ± 0.0002

Values with the same letters are not significantly different (*P* < 0.05).

NS indicates lack of significant difference in CK concentrations between all time points.

**Figure 1:**
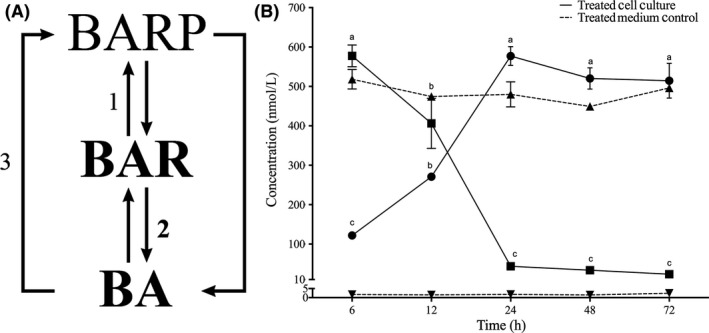
A, Observed benzyladenine metabolism demonstrating the structural derivatives (nucleotide, N6‐benzyladenine‐9‐riboside‐5′ (either mono‐, di‐, or tri‐) phosphate; riboside (BARP), benzyladenine riboside (BAR); free base (FB), and benzyladenine (BA) detected in the treated HeLa culture supernatants and the proposed enzymes responsible for the interconversions between cytokinin (CK) structural forms. This information was inferred from this study and other in vitro mammalian cell culture investigations.^30^, ^31^, ^50^, ^51^, ^55^ Numbers represent inferred enzymes as follows: 1. adenosine kinase (EC 2.7.1.20); 2. purine nucleotide phosphorylase (EC 2.4.2.1); 3. adenine phophoribosyltransferase (EC 2.4.2.1). The remaining arrows demonstrate alternative conversions that can occur via specific enzymes in other CK producing organisms, such as *Arabidopsis*.^5^, ^6^, ^8^, ^9^, ^19^ Bolded text refers to the CK forms that were dominant and the proposed enzyme activity that we observed in this study. B, Conversion of BAR (■) into the FB fraction, BA (●) by HeLa cells treated with 10^−6^ mol/L BAR throughout a 72‐h incubation period indicated by a solid line. A 10^−6^ mol/L BAR medium (no cell) control was incubated throughout this same period showing no breakdown of the compound over time, indicated by dashed lines and triangles (BAR, ▲; BA, ▼). The hormone concentrations were determined using high‐performance liquid chromatography‐positive electrospray ionization tandem mass spectrometry Presented values are means ± SE (n = 3). A separate ANOVA was performed for each CK form for the treated medium control and the treated cell culture supernatants with time as the dependent variable, followed by Tukey's post hoc analysis. Significant differences in CK concentration were found only in the treated cell cultures, where the same letters indicate values that are not significantly different (*P* < 0.05). Values are followed by letters only when significant differences were detected

The supernatant samples of the 10^−9 ^and 10^−6 ^BAR‐supplemented cultures were dominated by the R and FB forms of BA (Figure 1A). A clear trend was observed in the concentration of both BAR and BA throughout the culture incubation period—BAR steadily decreased while the levels of BA steadily increased, indicating the HeLa cells were capable of taking up the BAR from the medium and converting the Rs into its FB form, BA (Figure 1B; Table 3). As previously mentioned, no natural breakdown/conversion of BAR into BA was observed in the cell‐free, 10^−6 ^mol/L BAR‐supplemented medium blank, further emphasizing the active role of HeLa cells in converting exogenous BAR to BA (Figure 1B). In the 10^−6 ^M BAR‐supplemented cultures, significant twofold increases in the FB, BA, concentration were found between the 6‐ and 12‐hour time points, and the 12‐ and 24‐hour time points (Figure 1B; Tukey's *P* < 0.05). Interestingly, an opposite trend was observed in BAR levels detected in the HeLa supernatant. A significant difference in the Rs, BAR, concentration was found between the 12‐ and 24‐hour time points, for which a nine‐fold decrease in concentration was noted. While supernatant samples from the 10^−9 ^mol/L BAR‐treated cultures had a similar inverse relationship between BAR and BA levels, significant differences were only observed for BA concentrations between the 6‐ and 72‐hour time points (Table 3; Tukey's *P* < 0.05). Furthermore, there were no significant differences between the concentrations of any BA‐type CKs when cultures were treated with the lowest BAR concentration (10^−12 ^mol/L BAR; data not shown). These results suggest that the supplementation of 10^−6^ mol/L BAR was the most effective in revealing the significant changes in hormone profiles in HeLa supernatants.

For both the untreated and BAR‐treated supernatant samples, iP levels increased throughout the incubation period—coinciding with the increase in cell density—while the levels of iPR remained relatively low and unchanged between all treatments and sampling times (Table 2, Table 3). This suggests BAR treatment does not affect endogenous iP secretion by the HeLa cells.

Interestingly, treatment with BAR at the 10^−9^ and 10^−6 ^mol/L concentrations resulted in the highest detected CK levels in the pellet samples. iPRP dominated the pellet hormone profiles accounting for 91% and 93% of the total CKs concentrations in the 10^−9^ and 10^−6 ^mol/L BAR‐treated pellet samples respectively (Table 3). By contrast, the levels of iPRP in the untreated HeLa pellet accounted for 45% of the total CK concentrations (Table 2). Upon treatment with 10^−6 ^mol/L BAR, the levels of endogenous iPRP in the cell pellet increased by threefold respectively, compared to the untreated HeLa pellet samples (Table 2 & 3).

## 4 DISCUSSION

The great majority of knowledge about CK function and metabolism comes largely from studies involving plant taxa.^1^, ^3^ While CKs have been discovered in all kingdoms of life (with the exception of Archaea), information on their biosynthesis and role in the animal kingdom is still very limited. By using mass spectrometry, this study reports the presence of several additional mammalian CK types beyond the ones published to date,^22^, ^32^, ^37^ and documents the fate of iP‐ and 2MeS‐type CK production in HeLa cell culture. Moreover, this study reveals the interconversion of iP‐type CKs consistent with findings in plants, fungi, and other CK‐producing organisms.^4^, ^16^, ^38^ Similarly, we demonstrate that HeLa cells are able to take up and metabolize an exogenously applied CK, BAR, converting it into different structural derivatives.

### 4.1 Novel mammalian CK forms detected

In this study, the presence of iP‐type and 2MeS‐type CKs was detected. Formerly, N^6^‐isopentenyladenosine, denoted i^6^A, was the only known mammalian CK, and it was determined to exist at position 37 of tRNA molecules, which is a position that can bind codons starting with uridine.^37^ Recently, six additional CK forms were newly identified from 18 different canine tissue samples: iPR (synonymous with i^6^A), iPRP, *c*ZR, *c*ZRP, 2MeSiP, 2MeSiPR, and 2MeSZR.^32^ Here, we report the nucleotide form and FB forms of iP, which have never been previously documented as endogenously produced compounds in mammalian cell culture. Furthermore, we document the presence of the 2MeS CK forms, 2MeSiP, 2MeSiPR, 2MeSZ, and 2MeSZR in HeLA cells. Our results align with the recently identified CK forms in canine tissues and collectively pose many questions about the role of CK biosynthesis and metabolism in mammalian systems.

Regarding the biosynthesis of the detected iP‐type and 2MeS‐type CKs in this study, there are two key enzymes responsible for the production of these molecules. In humans, the tRNA‐isopentenyltransferase 1 (TRIT1; EC 2.5.1.75) enzyme is responsible for the modification of the A37 position in specific mitochondrial and cytosolic tRNA molecules,^39^ thus allowing for the synthesis of iP‐type CKs. This modification is evolutionarily ancient, and the resulting molecule is denoted i^6^A37‐tRNA. This subset of tRNA molecules can be further modified through methylthiolation to form ms^2^i^6^A37‐tRNAs. The ms^2^ modifications have been documented in bacteria and mammals.^28^ In humans, cyclin‐dependent kinase 5 regulatory subunit‐associated protein 1 (CDK5RAP1) is responsible for the methylthiolation of i^6^A37 to ms^2^i^6^A37.^40^, ^41^ While these studies provide the basis for the synthesis of 2MeS‐type CKs in mammals, they do not classify the resulting ms^2^i^6^A37‐tRNAs as a CK. Regardless of classification, the aforementioned studies imply that the necessary enzyme systems are present to produce the dominant CK forms that were detected in this work.

It should be noted that the culture medium contained trace levels of CKs, specifically iPR, 2MeSZ, and 2MeSiP. Prior to this research, a selection of common mammalian cell culture media (DMEM, MEM, and RPMI) were tested for exogenous CK presence, none of which were free of CKs. Of the various CK forms detected in common cell culture media, iPR, 2MeSZ, and 2MeSiP were the three analytes most consistently found. In some instances, the same medium from different manufacturer's batches yielded different CK profiles. This can be attributed to the specific organic ingredients required in medium formulations, such as yeast extract which is known to contain CKs.^42^ While mammalian cells have the machinery to synthesize 2MeS‐type CKs,^28^ the presence of these CKs in the medium disallows conclusions about the source of biosynthesis of these molecules. Importantly, however, the presence of iP, iPRP, 2MeSiPR, and 2MeSZR were detected only upon addition of HeLa cells to the medium.

### 4.2 HeLa CK metabolism

The structural derivative of the CK determines the biological activity of the molecule.^1^, ^11^ The conversion of CK precursors into various CK types and their structural derivatives requires many distinct enzymes. In this study, in the cell pellet and supernatant samples, we detected: nucleotide (RPs), Rs, FBs, and 2MeS CK forms. The presence of these various forms indicates that the HeLa cells have the enzymes responsible for facilitating the conversion of CKs into their various structural derivatives. Such conversions and enzymes are well‐documented in plants.^3^, ^19^ Based upon the reports of CK biosynthesis in plants and other CK‐producing organisms, we highlight that several enzymes that may be responsible for the interconversions of CKs that we observed.

The FB, iP, was detected in the highest concentrations in the HeLa culture supernatants, while the highest concentrations of the nucleotide form of iP, iPRP, were found in the cell pellets. By contrast, the riboside form of iP, iPR (i^6^A in humans), was present at low levels in the HeLa culture supernatants. These results follow the characteristic trends observed for microbial in vitro cultures, where RPs are retained within the cell pellet and the FB fractions are secreted outside of the cell, thus accumulating in the supernatant.^43^ As previously stated, the TRIT1 enzyme facilitates the synthesis of the tRNA‐bound form of iPR.^22^ Derived from tRNA turnover, iPR can be found in mammalian systems in its unbound form.^27^ Early reports indicate that these molecules are then rapidly metabolized and excreted in the urine.^44^, ^45^ However, the detection of the nucleotide fraction of iP, iPRP, within the cell pellet, as well as the other FB, R, and 2MeS CK forms suggests that the complexity of mammalian CK production and metabolism is greater than previously understood. Furthermore, in the recent report on CK profiles in canine tissues, a tissue‐ and organ‐specific trend was observed, which to some extent resembles the organ‐specific CK dynamics in plants.^32^, ^46^, ^47^ Coupling these new findings about mammalian CKs, we suggest there is a greater role for the breakdown product of iPR‐tRNA turnover as is evidenced by the numerous CK forms detected both intracellularly and extracellularly.

A variety of enzymes are necessary to facilitate the biological conversions that result in the production of the various CK forms reported in this study. It is known that iPR occurs naturally in mammalian systems as a result of tRNA turnover. Therefore, iPR, as a starting point substrate, is used to describe the proposed enzymes responsible for the detected CK forms in this study. To convert iPR into the FB form, iP, an enzyme is necessary to cleave the ribose moiety from the adenine molecule. In plant systems, this enzyme is known as adenosine nucleosidase EC 3.2.2.7, also referred to as uridine‐ribohydrolase in *Arabidopsis thaliana*.^48^ More recently, an additional enzyme capable of facilitating the same reaction from CK riboside to FB was identified in *Solanum tuberosum *(potato)—referred to as purine nucleoside phosphorylase (EC 2.4.2.1).^49^ While there are currently no known nucleoside hydrolases in mammals, purine nucleoside phosphorylases (PNPs) have been characterized in mammalian systems.^50^, ^51^, ^52^ Therefore, we propose this enzyme may be responsible for the conversion of iPR into iP in HeLa cells as observed in this study. However, there has been no established role of PNPs in CK conversions in mammals to date. To explain the presence of iPRP in the HeLa cell pellets, we propose that the enzyme, adenosine kinase EC 2.7.1.20, previously characterized in mammals,^53^ is responsible for the intracellular phosphorylation of iPR into iPRP. Adenosine kinase has been shown in numerous in vitro mammalian studies to be responsible for phosphorylation of CK ribosides into CK nucleotides,^30^, ^31^, ^54^, ^55^ and more recently documented in fungi.^56^ An alternative pathway for synthesis of CK nucleotides in plants is possible through the enzyme adenine phosphoribosyltransferase (APRT; EC 2.4.2.7), which converts FBs directly into the nucleotide fraction.^57^, ^58^ APRT has been documented in mammalian systems where it is capable of converting purine bases into their respective monophosphates.^54^, ^55^, ^59^ Specifically, APRT is involved in the purine nucleotide salvage pathway, where it catalyzes the addition of a phosphoribosyl group from the phosphoribosyl pyrophosphate (PRPP) molecule to adenine, thus forming AMP and releasing pyrophosphate (PPi).^60^ Therefore, this study supports previous findings that APRT is active in mammalian cells, facilitating conversions from the nucleotide form of CKs directly into the FB form.

### 4.3 BAR treatment & metabolism

N^6^‐benzyladenosine is an aromatic CK composed of a benzyl ring side chain at the N^6^ position of the adenine. BAR was originally thought to be a synthetic CK, as it was not detected and identified in natural plant tissues.^7^ However, it was eventually isolated and identified along with its metabolites in various plant species.^8^, ^61^, ^62^ Interestingly, in plant assays, BAR and its derivatives have been found to be very active.^29^ Moreover, in human cancer cell lines, BAR exhibits potent anticancer activity against a wide range of cell lines at micromolar concentrations.^30^ Furthermore, many other CKs have been tested and show similar cytotoxic effects on a wide range of cancer cell lines. These studies indicate that the potent dose‐dependent cytotoxic effects are linked to a variety of processes: intracellular phosphorylation of the CKs leading to a depletion of ATP and genotoxic stress, among others.^54^, ^63^, ^64^ The findings relating to intracellular phosphorylation of exogenously applied CKs support the results of the current study as they indicate mammalian cells are capable of converting exogenously applied CKs into nucleotide forms.

N^6^‐benzyladenine and its structural derivatives have been used extensively in plant studies as BA is known as one of the most active and affordable CKs to synthesize.^7^ Furthermore, this CK is easily labeled at multiple different positions making it a good candidate molecule for tracking; however, because BA has not been detected in HeLa cells, its labeling was not necessary for its use as a metabolic tracking molecule for the purposes of our study. Owing to its high biological activity in assays, BA and its structural derivatives gained attention in mammalian systems. FB CK forms have been shown to induce cell differentiation at concentrations between 25‐100 µmol/L in leukemia cell lines, whereas riboside forms cause apoptosis of leukemia cell lines at much lower µmol/L concentrations.^65^, ^66^ In the current study, there were no noticeable changes in morphology or cell density between the three different BAR concentrations as observed upon the trypan blue staining. Given the cytotoxic nature of the riboside fraction, it was expected that the 10^−6 ^mol/L concentration would be the most likely to induce cytotoxicity; however, this effect was not observed. As previously mentioned, apoptosis induced by various N^6^ compounds is related to the intracellular phosphorylation of those compounds and activation of caspases.^30^, ^31^, ^54^, ^55^ Unlike previous studies, the nucleotide fraction of BAR, BARP, was not the dominant metabolite following treatment with BAP. BARP was only detected at low levels in the 10^−9^ or 10^−6 ^mol/L BAR‐treated HeLa pellet samples, and these levels were consistent with that found in the 10^−6 ^mol/L BAR‐treated, no‐cell control medium indicating no direct conversion of BAR into its nucleotide metabolite, BARP. Instead, we observed a clear conversion of BAR into the FB form, BA. We propose that the enzyme responsible for converting ribosides into FBs in HeLa cultures is purine nucleoside phosphorylase. It appears that the HeLa cells can avoid the cytotoxicity of BAR by an alternative mechanism through which BAR is converted into the FB form, BA, instead of the nucleotide form, BARP. This finding is unique to this study and is contrary to what has previously been documented in mammalian cells treated with CKs.^30^, ^31^, ^54^ Interestingly, a large increase in the concentration of iPRP within the BAR‐treated cell pellets was observed compared to the untreated HeLa pellets.

This study reports the detection of novel CK forms, not previously identified in mammalian cell culture. Furthermore, we demonstrate the ability of HeLa cells to take up and metabolize the naturally occurring and exogenously supplied sources of CKs. Based on our data utilizing a single mammalian cell line, we recommend examining the metabolism of CKs using multiple different cell lines from various tissue origins to expand our knowledge on endogenous CK metabolism within mammalian systems. As it stands, our findings are limited to a single cell line; therefore, additional studies in various mammalian cell lines will lay the basis for CK metabolism in mammals. Furthermore, the proposed enzymes responsible for the interconversion of CKs observed in this study need to be validated in future studies to confirm whether they play a role in CK metabolism in mammalian systems.

## CONFLICT OF INTEREST

The authors declare no conflict of interest.

## AUTHOR CONTRIBUTIONS

Conceptualization, Megan M. Aoki, Mark Seegobin, Anna Kisiala, Adam Noble, Craig Brunetti, and R. J. Neil Emery; Data curation, Megan M. Aoki and Mark Seegobin; Funding acquisition, Craig Brunetti and R. J. Neil Emery; Investigation, Megan M. Aoki and Anna Kisiala; Methodology, Megan M. Aoki, Mark Seegobin, Anna Kisiala, Adam Noble, Craig Brunetti, and R. J. Neil Emery; Writing, original draft, Megan M. Aoki, Mark Seegobin, Anna Kisiala, Craig Brunetti, and R. J. Neil Emery; Writing, review & editing, Megan M. Aoki, Mark Seegobin, Anna Kisiala, Adam Noble, Craig Brunetti, and R. J. Neil Emery.
