# Supplementary material for: Phytohormone metabolism in human cells: Cytokinins are taken up and interconverted in HeLa cell culture
Source: FASEB Bioadv. 2019 Mar 12;1(5):320–31. doi: 10.1096/fba.2018-00032 (PMC6996375; doi:10.1096/fba.2018-00032)
Supplement: Supplementary file 1 [file FBA2-1-320-s001.pdf]

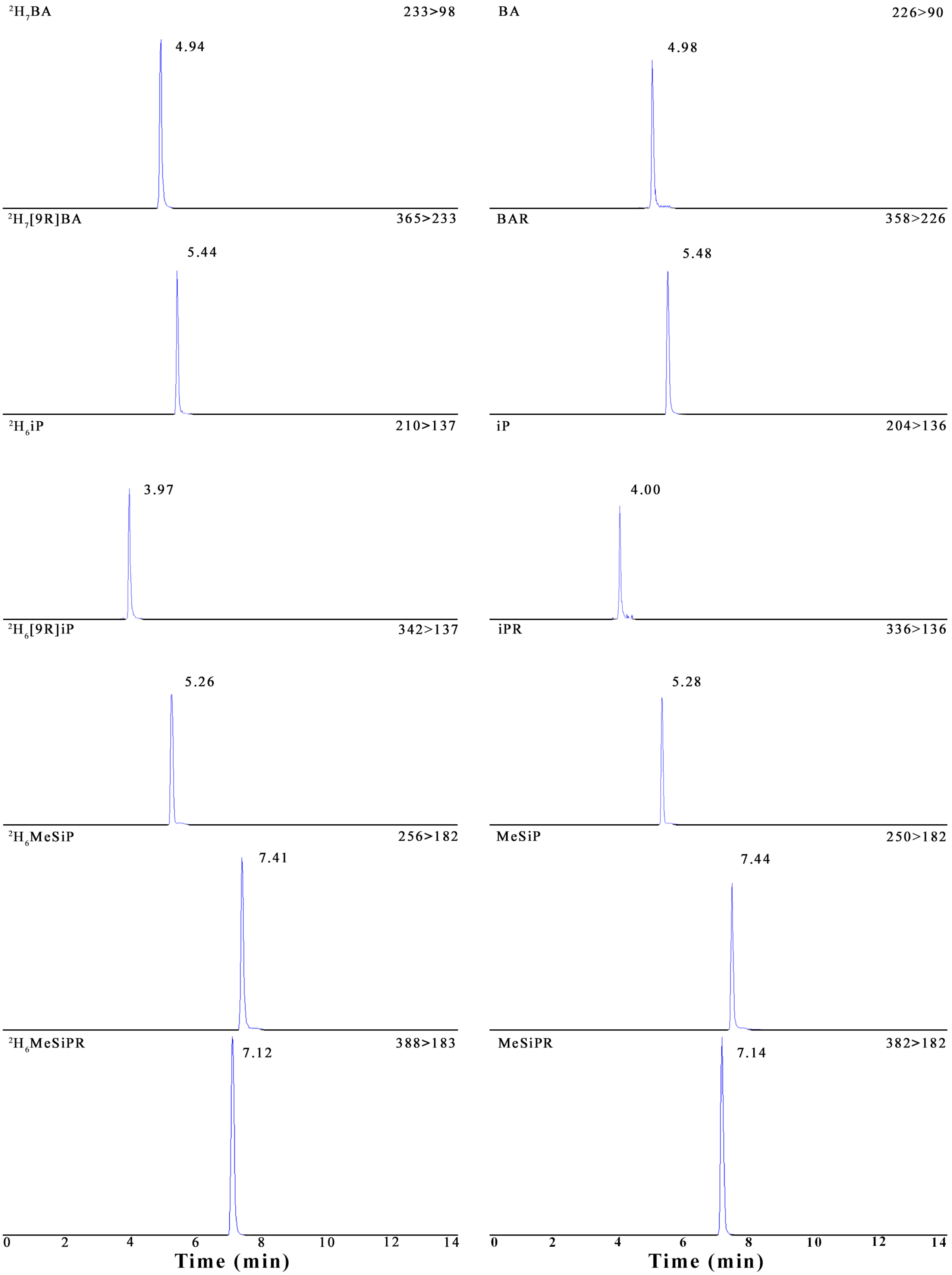

FIGURE S1. QTRAP® 5500-derived representative chromatograms for selected CK analytes using the described CK extraction and quantification methods of HeLa supernatant extracts. The labeled standard for each analyte of interest is shown on the left panel, and the corresponding unlabeled analyte is shown on the right panel.
